# Influences of cancer symptom knowledge, beliefs and barriers on cancer symptom presentation in relation to socioeconomic deprivation: a systematic review

**DOI:** 10.1186/s12885-015-1972-8

**Published:** 2015-12-23

**Authors:** Grace M. McCutchan, Fiona Wood, Adrian Edwards, Rebecca Richards, Kate E. Brain

**Affiliations:** Institute of Primary Care and Public Health, School of Medicine, Cardiff University, Neuadd Meirionnydd, Heath Park, Cardiff, CF14 4YS UK

**Keywords:** Patient delay, Symptom knowledge, Cancer beliefs, Barriers to symptom presentation, Socioeconomic status

## Abstract

**Background:**

People from lower socioeconomic groups have worse survival outcomes for cancer, which in part reflects later-stage disease at diagnosis. The mechanisms underlying delayed cancer symptom presentation in lower socioeconomic groups are not well understood.

**Methods:**

Systematic review of studies of actual or anticipated symptom presentation across all tumour sites. Included studies measured socioeconomic group, symptom presentation and one or more of the following variables: cancer symptom knowledge, beliefs about cancer, barriers/facilitators to symptom presentation.

**Results:**

A total of 60 studies was included. Symptom knowledge overall was lowest and actual presentation time was longest in lower socioeconomic groups. Knowledge for specific symptoms such as lumps and bleeding was good and encouraged timely symptom presentation, in contrast to non-specific symptoms which were not well recognised. The combination of fearful and fatalistic beliefs was typically associated with later presentation, especially in lower socioeconomic groups. Emotional barriers such as ‘worry what the doctor might find’ were more frequently reported in lower socioeconomic groups, and there was evidence to suggest that disclosing symptoms to family/friends could help or hinder early presentation.

**Conclusions:**

Poor symptom knowledge, fearful and fatalistic beliefs about cancer, and emotional barriers combine to prolong symptom presentation among lower socioeconomic groups. Targeted interventions should utilise social networks to improve knowledge of non-specific symptoms, challenge negative beliefs and encourage help-seeking, in order to reduce avoidable delays and minimise socioeconomic group inequalities.

**Electronic supplementary material:**

The online version of this article (doi:10.1186/s12885-015-1972-8) contains supplementary material, which is available to authorized users.

## Background

Socioeconomic inequalities in cancer survival outcomes exist, but the reasons for this are not fully understood [1–3]. Survival differences are likely to reflect later-stage disease at diagnosis [2, 4, 5] partly as a consequence of delayed cancer symptom presentation in people from lower socioeconomic groups [6]. By eradicating socioeconomic inequalities at stage of diagnosis, it is estimated that 5600 patients in the UK annually could be diagnosed with earlier stage disease [7], and that 11 % of deaths from cancer could be avoided if three-year survival in lower socioeconomic groups matched that in higher socioeconomic groups [1].

**Fig. 1:**
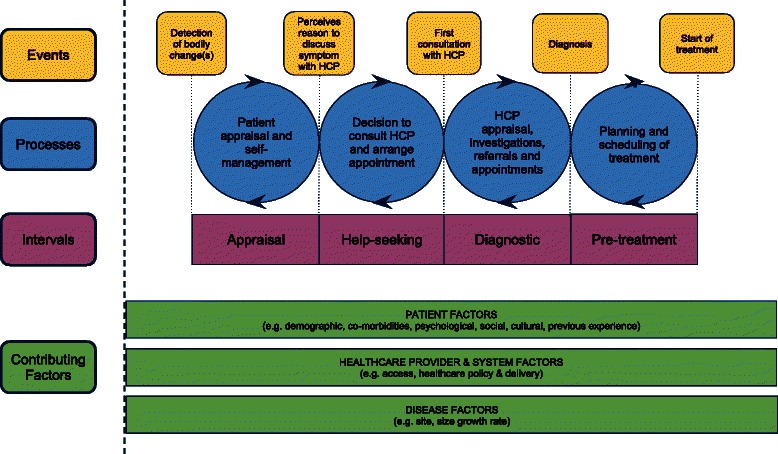
Model of pathways to treatment. Produced with permission of SAGE Publications Ltd., London, Los Angeles, New Delhi, Singapore and Washington DC, from Walter FM, Scott SE, Webster A, Emery JD. ‘The Andersen Model of Total Patient Delay: a systematic review of its application in cancer diagnosis’. J Health Services Research & Policy (© Walter, 2012)

‘Patient delay’ is defined as the time between discovery of a cancer symptom and the initial visit to a healthcare professional. It accounts for the greatest proportion of delay time in the pathway from symptom discovery to the start of cancer treatment [8–10] and has been associated with socioeconomic deprivation [6]. Patient delay has been conceptualised in Walter et al*.*’s Model of Pathways to Treatment, with various stages involving an ‘appraisal interval’ during which the individual detects a bodily change, and a ‘help seeking interval’ in which the individual decides to seek medical help (see Fig. 1 [11]). Evidence suggests that knowledge of cancer symptoms is important during the appraisal stage, with potential misattribution of symptoms attenuating the decision to present [12, 13]. Beliefs about cancer are considered to be important in both the appraisal and help-seeking stages, where emotions such as fear might influence interpretation of symptoms [12] and the decision to seek medical help [6, 14–17]. Barriers such as competing life events and ease of getting a medical appointment are thought to delay symptom presentation during the help-seeking interval [11].

The contribution of socioeconomic and other demographic factors to delayed presentation has been highlighted in the Model of Pathways to Treatment, and more recently in the updated National Awareness and Early Diagnosis Initiative (NAEDI) framework designed to conceptualise the route from public awareness and beliefs about cancer to cancer survival outcomes ([18]). Empirical evidence supports associations between lower socioeconomic group and poor cancer symptom knowledge [19], fearful and fatalistic beliefs about cancer [20] and emotional barriers such as worry about what the doctor may find [19]. These findings help to explain why people from lower socioeconomic groups tend to present with more advanced stage cancers, and hence have worse survival outcomes [1–5]. However, a more detailed understanding of psychosocial influences on the relationship between socioeconomic deprivation and cancer symptom presentation is essential to developing behavioural interventions designed to promote timely presentation and reduce socioeconomic inequalities in cancer outcomes.

Attempts to understand why people might delay seeking medical help for cancer symptoms have examined actual or anticipated symptom presentation behaviour, exploring perceived barriers to symptom presentation. Prospective study designs are difficult due to follow-up of a large sample, so studies frequently use retrospectively recalled or hypothetically anticipated symptom study designs. Previous reviews have focused on tumour site-specific delay factors [15, 16, 21] or common cancers only [6], or have been restricted to qualitative studies [17] and patients with cancer [6, 16, 17]. The purpose of the current systematic review was to explore how knowledge, beliefs and barriers/facilitators to symptom presentation affect actual or anticipated cancer symptom presentation in relation to socioeconomic group and across all tumour sites.

## Method

Identification of included studies followed the PRISMA guidelines [22]. The protocol was registered on PROSPERO (CRD42014013220 [23]) and is available on the NIHR HTA programme website (www.hta.ac.uk). At all stages of the search, data extraction and quality appraisal, 10 % of studies were double checked for consistency by a second member of the research team (RR). All discrepancies were resolved through discussion.

### Search strategy

The literature was searched up to July 2015 on the electronic databases of MEDLINE, PsychINFO, EMBASE and CINAHL. The de-duplicate function was used on Ovid and CINAHL before reviewing abstracts. Manual searches of reference lists of included studies were performed. A SPIDER (Sample, Phenomenon of Interest, Design, Evaluation, Research type) search strategy tool was used for retrieval of studies (see Additional file 1: Appendix 1 [24]). Databases were searched using terms relating to *symptom presentation, cancer symptom knowledge, beliefs about cancer, perceived barriers and facilitators to symptom presentation* (see Additional file 1: Appendix 1)*.*

### Inclusion criteria

Publications that measured and reported data for symptom presentation and socioeconomic group were included. ‘Symptom presentation’ was defined as actual symptom presentation (retrospectively recalled) or anticipated symptom presentation (hypothetically estimated) measured as continuous (time to presentation) or binary (did/did not present) variables. ‘Socioeconomic group’ was defined in terms of individual level socioeconomic indicators including education, income, home/car ownership, occupation and employment, and/or area-level indicators based on postcode. In addition, publications were included if they measured and reported one or more of the following domains of interest:

- ‘Knowledge’: studies which assessed knowledge for the symptoms of cancer through recall e.g. ‘What symptoms of cancer can you list?’ or recognition methods e.g. ‘Which of these are symptoms of cancer?’, or through retrospective recall of symptom interpretation at the time of symptom discovery.
- ‘Beliefs’: studies which explored any positive (e.g. beliefs about the benefits of early diagnosis and curability) or negative (e.g. fear and fatalism) beliefs surrounding cancer.
- ‘Perceived barriers/facilitators’: studies which assessed any anticipated or actual barriers or facilitators to symptom presentation.

There were no restrictions on date of publication or study methodology. Only English language studies from high income countries as classified by Organisation for Economic Co-operation and Development (OECD) membership (OECD, 2014 [25]) were included.

### Exclusion criteria

**Fig. 2:**
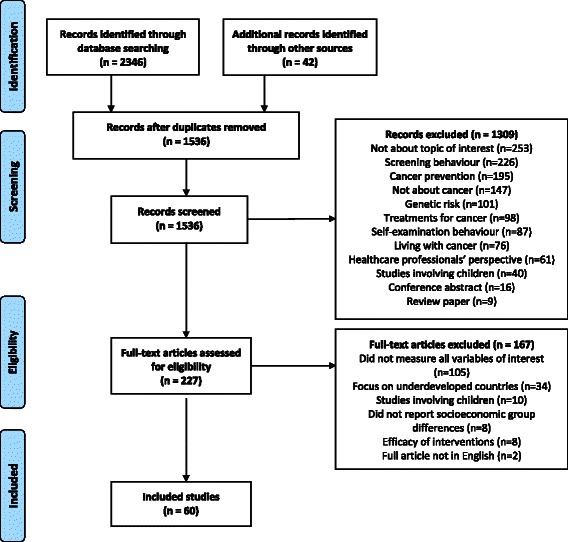
PRISMA flow diagram. Produced using a downloadable template available at http://www.prisma-statement.org/ (Moher et al, 2009 [22])

Studies that did not measure and report symptom presentation, socioeconomic group and one or more of the domains of interest were excluded. Studies not relating to cancer, and those examining screening behaviour, self-examination behaviour, efficacy of interventions, genetic risk, healthcare professionals’ perspective, cancer prevention, treatments for cancer or living with cancer and studies involving children were excluded. Studies from low/middle income countries, not written in English, review papers or conference abstracts were excluded (Fig. 2).

### Data extraction and synthesis

Data were extracted onto a template using the following headings: method, sample characteristics, tumour site, symptom presentation, knowledge, beliefs, perceived barriers/facilitators and socioeconomic group measure. A meta-analysis was precluded due to the heterogeneity of included studies and a narrative synthesis was performed [26].

### Critical appraisal

The methodological quality of all included studies was examined using the Critical Appraisal Skills Programme tool (CASP, 2014 [27]) appropriate for the study design. Quality was assessed according to each domain on the CASP checklists: rationale of study, methodology, design, recruitment, data collection, data analysis, ethical issues, reporting of findings and contribution to research*.* Overall quality was categorised as good, medium or poor.

## Results

The search returned a total of 1536 studies after 810 duplicates had been removed. A total of 1309 studies was excluded based on title and abstract, leaving 227 studies to be read in full. A total of 60 studies met the inclusion criteria (see Fig. 2). Eleven of these studies were found through hand searching reference lists.

Included studies employed qualitative methods (*n* = 15), quantitative methods (*n* = 42) and mixed methods (*n* = 3). Quality of studies was good (*n* = 18), medium (*n* = 37) and poor (*n* = 5). Limitations of lower quality studies included measuring but not reporting socioeconomic group differences for all outcome measures, leaving a long period of time between cancer diagnosis and participation in the study and recruitment of samples biased towards higher socioeconomic groups. The overall combined percentage agreement between raters (GM and RR) for inclusion/exclusion of studies, critical appraisal and data extraction was 87 %.

A total of 53 studies examined time to symptom presentation, seven studies reported presentation behaviour (if participants did or did not present or anticipate presenting to their doctor with reported symptoms), 45 studies measured actual symptom presentation, 15 studied anticipated symptom presentation, 46 studies assessed knowledge for cancer symptoms, 32 studies explored beliefs about cancer and 50 studies examined perceived barriers/facilitators to symptom presentation. The numbers of studies by tumour site were as follows: breast (*n* = 22), any cancer/multiple tumour sites (*n* = 15), colorectal (*n* = 7), skin (*n* = 6), oral and pharyngeal (*n* = 3), ovarian (*n* = 3), lung (*n* = 2), gynaecological (*n* = 1), and prostate (*n* = 1) (see Table 1). Results are presented according to domain headings.

**Table 1:** Table of included studies

Study	Method	Sample	Country	Tumour site	Socio-economic measure	Measures: Knowledge (K), Beliefs (B), Perceived barriers (PB), Perceived facilitators (PF), Symptomatic Presentation (SP)	Measure of association between variables of interest and socioeconomic indicator (qualitative studies not applicable)	Quality appraisal
Brain et al. (2014) [28]	Hypothetical Quantitative	1043 women. Aged 50 years and over	Wales	Ovarian	Postcode, education	K: Recognition (mean, 6.85 symptoms) B: Cancer worry PB: Emotional and practical barriers SP: Sought medical help in under 3 weeks (*n* = 898)	K: Lower education associated with lower knowledge (F(_2, 1005_) = 8.23, *p* < 0.001); higher deprivation (postcode) associated with lower knowledge (F_3,886_ = 2.82, *p* < 0.05) B: NR PB: NR SP: Higher education associated with longer time to SP, (OR = 2.64, p ≤ 0.001); NS difference between deprivation by postcode and anticipated delay (X^2^_(3)_ = 6.73, *p* > 0.05) NS	Good
Brouha et al. (2005) [76]	Retrospective Quantitative	189 men and women. Mean age: 59 years	Holland	Oral and Pharyngeal	Education, income	K: Symptom interpretation (‘cancer’, *n* = 2), misattribution of symptoms to dental problems delayed SP PB: Symptom did not interfere with daily life PF: Persistence of symptom, development of new symptom SP: Mean time to symptom presentation (pharyngeal, 45 days; oral, 28 days)	K: NR PB: NR PF: NR SP: Education and income not associated with time to SP (statistics NR)	Medium
Burgess et al. (1998) [45]	Retrospective Qualitative	185 women. Mean age: 54 years	UK	Breast	Occupation	K: Symptom interpretation (46 % thought their symptom indicated cancer) B: Fear PF: Symptom disclosure, appearance of new symptoms, appointment booked with GP for another reason SP: Waited over 3 months to seek medical help (19 %)		Medium
Burgess et al. (2000) [67]	Retrospective Qualitative	158 women. Mean age: 53 years	UK	Breast	Occupation	PB: Life events SP: Waited over 3 months to seek medical help (18 %)		Medium
Burgess et al. (2001) [43]	Retrospective Qualitative	46 women. Mean age: 54.1 years	UK	Breast	Occupation	K: Symptom interpretation (‘lump’ most attributed to cancer) B: Consequences of treatment PB: Not wanting to bother the doctor, poor health service utilisation, competing life priorities PF: Symptom disclosure, change in symptom SP: Waited over 3 months to seek medical help (*n* = 31)		Medium
Cameron and Hinton (1968) [58]	Retrospective Quantitative	83 women	UK	Breast	Education, husband’s occupation	K: Symptom interpretation B: Fear, worry SP: 61 % sought medical help within 1 month	K: NR B: NR SP: Higher education associated with shortest time to SP for lump symptoms (*x*^2^ = 6.6, *p* < 0.05); Higher social group (husband’s occupation) associated with shortest time to SP (*x*^2^ = 3.02, *p* < 0.01)	Poor
Caplan (1995) [44]	Retrospective Quantitative	162 women	US	Breast	Income, education, employment	PB: Fluctuating symptoms, relationship with GP SP: Waited over 2 months to seek medical help (*n* = 27)	PB: NR SP: Lower socioeconomic group (various indices) associated with longer time to SP, but NS: High vs low income (OR 2.56, 95 % CI: 0.68-8.64*); High vs low education (OR 1.07, 95 % CI: 0.41-2.77*); Working vs non-working (OR 0.72, 95 % CI: 0.27-1.99*)	Poor
Carter-Harris et al. (2015) [69]	Retrospective Qualitative	11 men (*n* = 4) and women (*n* = 7). Age range: 40-76 years	US	Lung	Education, employment	K: Symptom interpretations (one participant was alarmed at symptoms) PB: Vague and intermittent nature of symptoms PF: Worsening of symptoms, good relationship with GP SP: Immediate (*n* = 1)		Medium
Chonjnacka-Szawlowska et al. (2013) [36]	Retrospective Quantitative	301 men (*n* = 186) and women (*n* = 115). Mean age: 42.3 years	Poland	All	Education	K: Recall, mean: 1.51 B: Fatalism and cancer curability SP: Mean time to symptom presentation: 6 months and 10 days; stage of cancer	K: NR B: NR SP: NS correlation between education and stage of cancer (statistics NR)	Medium
Coates et al. (1992) [42]	Retrospective Quantitative	735 women (410 black and 325 white). Age range: 20 to 79	US	Breast	Education, occupation, poverty index (income/no of people in household)	K: Symptom interpretation B: Fatalism PB: Symptom disclosure, other comorbid conditions, appointment with doctor booked for another reason SP: Median time to symptom presentation (black women, 16 days; white women, 14 days)	K: NR B: NR PB: NR SP: Higher education associated with shorter time to SP (Mantel-cox 1.43, 95 % CI: 1.11-1.86, *p* < 0.05); Low deprivation (poverty index) associated with shorter time to SP (Mantel-Cox 1.24, 95 % CI: 1-1.54, *p* < 0.05)	Good
Cockburn et al. (2003) [54]	Retrospective Quantitative	1332 men (40 %) and women (60 %). Aged 40 years and over	Australia	Colorectal (Bowel)	Education	K: Recall (25 % could not recall any symptoms), symptom interpretation B: Benefits of early diagnosis SP: 306 had experienced a symptom, 31.9 % did not seek medical help	K: Higher education associated with higher K of symptoms (PR 0.93, 95 % CI: 0.89-0.96*) B: Higher education more likely to hold positive beliefs about the benefits of early diagnosis (statistics NR) SP: NR	Medium
Esteva et al. (2013) [70]	Retrospective Quantitative	795 men (*n* = 489) and women (*n* = 291)	Spain	Colorectal	Social class, education	K: Symptom interpretation (‘not serious’, 65.6 %) PF: Symptom disclosure, good relationship with GP (trust) SP: Median time to symptom presentation (19 days)	K: NR SP: NS association between social class and time to SP (statistics NR), NS association between education and time to SP (statistics NR)	Medium
Facione and Facione (2006) [59]	Retrospective Qualitative	28 women. Mean age: 42.34 years	US	Breast	Income, education, health insurance	K: Symptom interpretation B: Fear, fatalism, benefits of early diagnosis PB: Worry about losing relationship with partner if diagnosed with cancer PF: Symptom disclosure SP: Sought medical help after 3 months (*n* = 15)		Medium
Facione et al. (2002) [56]	Hypothetical Quantitative	669 women. Mean age: 46.95 years	US	Breast	Income, education, health care insurance	K: Recognition (10 % recognised all or all but one symptoms) B: Fatalism PB: Difficulties with access, prejudice in health care, concerns about deportation, use of alternative therapies SP: Likely to delay (23.7 %).	K: Higher education associated with higher symptom recognition (F_3,690_ = 32.32, *p* < 0.001) B: NR PB: NR SP: Lack of insurance associated with longer time to SP (Cramer’s V = 0.187, *p* < 0.001); Lower education associated with longer time to SP (Cramer’s V = 0.288, *p* < 0.001); Lower income associated with longer time to SP (Cramer’s V = 0.291, *p* < 0.001)	Good
Facione et al. (1997) [84]	Hypothetical Quantitative	352 African American or Black women. Mean age: 38.6 years	US	Breast	Income, Education, Employment	B: Fear, fatalism PB: Poor health service utilization SP: 11.6 % = strong disposition to SP.	B: NR PB: NR SP: Stronger disposition to SP associated with lower education (r = 0.19, *p* < 0.01) and lower income (r = 0.32, *p* < 0.001)	Medium
Facione and Dodd (1995) [83]	Retrospective Qualitative	39 women. Mean age: 49.6 years	US	Breast	Income, education	K: Symptom interpretation B: Fear PB: Competing life priorities PF: Appearance of new symptom, worsening of symptoms, symptom disclosure SP: 59 % sought medical help within 1 week		Medium
Fitzpatrick et al. (1998) [57]	Hypothetical Quantitative	280 men. Mean age: 53.7 years	Ireland	Prostate	Health insurance, occupation	B: Fear PB: Poor health service utilisation, dislike of doctors, embarrassment SP: 81 % would seek medical help if developed urinary symptoms	B: NR PB: NR SP: Non-manual social class associated with higher willingness to attend GP with symptoms (OR 1.8, *p* < 0.05**)	Good
Forbes et al. (2011) [29]	Hypothetical Quantitative	1515 women from various ethnic groups (White, South Asian, Black). Aged 30 years and over	UK	Breast	Postcode (IMD)	K: Recognition (18 % recognised 5 or more non-lump symptoms) PB: self-efficacy, worry what the doctor might find, embarrassment, worry about wasting doctors time, difficulty getting an appointment SP: 73 % would seek help within 1 week	K: Differences between ethnic groups for cancer awareness not due to IMD score or lower level of education (statistics NR) PB: Differences between ethnic groups for PB not due to IMD score (statistics NR) SP: NR	Good
Forbes et al. (2014) [64]	Retrospective Quantitative	1999 men (*n* = 1077) and women (*n* = 922). Aged 50 or over	UK	All	Postcode	K: Symptom interpretation PB: 48 % of patients reported at least one barrier SP: Delay over 3 months (*n* = 21 %)	B: NR PB: NR SP: Lowest socioeconomic group associated with longest time to SP (1.51, 95 % CI: 1.18-1.88*)	Good
Freidman et al. (2006) [38]	Retrospective Quantitative	124 women. Mean age: 44.3 years	US	Breast	Employment, education	B: Fear PB: Worry what the symptom might be, difficulty getting an appointment, cost, denial SP: Mean time to symptom presentation (9 months)	B: NR PB: NR SP: Lower education associated with longest time to SP (Fishers Exact test, *p* < 0.01**)	Medium
Goldsen et al. (1957) [61]	Retrospective Quantitative	727 men and women	US	All	Income, education and occupation	K: Symptom interpretation (20 % thought symptoms indicated cancer) B: Cancer worry, fatalism PB: Poor health service utilization, symptom not noticed PF: Symptom disclosure SP: 51.3 % sought medical help under 30 days	K: NR B: NR PB: NR PF: NR SP: Lower income, education and occupation associated with longest time to SP (statistics NR)	Medium
Gould et al. (2010) [39]	Retrospective Qualitative	14 women. Aged range: 30 to 69 years	Canada	Breast	Education, employment, income	K: Symptom interpretation (poor for non-lump symptoms) B: Fear PB: Previous benign disease, watchful waiting, competing life priorities PF: Symptom disclosure, already have another appointment booked. SP: All women waited 8+ weeks		Medium
Grant et al. (2010) [82]	Retrospective Qualitative	15 men (*n* = 7) and women (*n* = 8). Aged 45 years and under	Scotland	Oral	Postcode	K: Symptom interpretation PB: Self-medication PF: Already had an appointment booked SP: Sought medical help within 8 weeks (*n* = 8)		Medium
Greer (1974) [68]	Retrospective Quantitative	160 women with stage I or stage II cancer. Aged 70 years and under	UK	Breast	Social Class	K: Symptom interpretation B: Fear, fatalism PB: Embarrassment SP: 64 % sought medical help within 1 month	K: NR B: NR PB: NR SP: NS difference between time to SP and social class (statistics NR)	Poor
Hunter et al. (2003) [30]	Hypothetical Quantitative	546 women. Mean age: 47 years	UK	Breast	Occupation	K: Recognition (good, mean 6.65) B: Beliefs about treatment SP: 58.6 % would seek immediate medical help.	K: NR NR: NR SP: Socioeconomic group not associated with time to SP (F_(1,518)_ = 0.29, *p* > 0.05)	Medium
Kakagia et al. (2013) [34]	Retrospective Quantitative	513 men (*n* = 56.5 %) and women (*n* = 43.5 %). Mean age: 67.5 years	Greece	Skin	Education, ethnicity, area of residence	K: Symptom interpretation B: Fear, fatalism PB: Other serious comorbidities, poor health service utilisation, dislike of doctors and hospitals, transport issues, worry about wasting doctors time, embarrassment, competing life demands PF: Symptom disclosure, active encouragement to seek medical help SP: Mean time to symptom presentation (3.9 months)	K: NR B: NR PB: NR PF: NR SP: Longer time to SP associated with lower socioeconomic group (OR 1.89, 95 % CI: 0.9-3.8. *p* < 0.001) and lower education (OR 3.01, 95 % CI: 1.6-5.6, *p* < 0.001)	Medium
Lam et al. (2009) [63]	Retrospective Qualitative	37 women. Age range 20-81 years	Hong Kong	Breast	Employment, education	K: Symptom interpretation B: fear, fatalism PB: Watchful waiting, poor general health service utilisation, cost, competing life priorities, embarrassment PF: Persistence of symptoms, appearance of new symptom, symptom disclosure, symptom interfering with daily life, appointment booked for another reason SP: Waited over 3 months to seek medical help (*n* = 14)		Medium
Li et al. (2012) [65]	Retrospective Quantitative	425 women. Mean age: 51.97 years	Hong Kong	Breast	Employment, education	B: Fear PB: Cost, gender of doctor, unsure where to seek medical help, competing life priorities, no history of breast problems, symptom disclosure PF: Symptom disclosure SP: Median time to symptom presentation (14 days)	B: NR PB: Symptom disclosure for women with lower education less likely to translate into immediate SP (*x*^2^ = 6.4, d.f. = 2, *p* < 0.05) PF: NR SP: Longer time to SP associated with higher education (OR 3.35, 95 % CI:1.19-9.42, *p* < 0.05) and full time employment (OR 2.52, 95 % CI: 1.18-5.36, *p* < 0.05)	Good
Loehrer et al. (1991) [71]	Retrospective Qualitative	128 men (*n* = 33) and women (*n* = 95). Mean age: 63 years	US	All	Employment, income, education	B: Curability of cancer, cancer is contagious, surgery causes cancer to spread SP: Poor for non-specific symptoms		Medium
Low et al. (2013) [31]	Hypothetical Quantitative	1000 women. Mean age: 47 years	UK	Ovarian	Education, car ownership, home ownership	K: Recall (poor, mean 0.6) and recognition (good, mean 6.3) PB: Mean number of barriers endorsed (2.2), emotional, practical and service barriers SP: Varied by symptom, most would seek help under 2 weeks	K: NR PB: NR SP: Higher socioeconomic group associated with longer time to SP (beta = 0.12, SE 0.05, *p* < 0.001**)	Good
Magarey et al. (1977) [72]	Retrospective Quantitative	64 women. Age in years: less than 40 (*n* = 13), 40-60 (*n* = 28), over 60 (*n* = 23).	Australia	Breast	Education	PB: Denial, anxiety SP: Most sought medical help within 2 weeks (*n* = 35)	PB: NR SP: Education not associated with time to SP (statistics NR)	Poor
Marlow et al. (2014) [78]	Hypothetical Qualitative	54 women from ethnic minority groups living with a comparison of white women. Age range: 25-64 years	UK	Breast and Ovarian	Employment, education, living arrangement	K: Recall (good for lumps/ bleeding, poor for other symptoms) B: Fear, fatalism, benefits of early diagnosis PB: Poor relationship with GP, emotional barriers, practical barriers, service barriers, competing life priorities PF: Symptom disclosure SP: Varied: days to months. All sought help within 3 months.		Medium
McCaffery et al. (2003) [50]	Hypothetical Quantitative	1637 men (*n* = 763) and women (*n* = 874). Age range: 16-74 years	UK	Colorectal	Education	K: Recall (poor) B: Fear SP: 92.8 % would anticipate seeking medical help if noticed blood in stool for more than 2 weeks.	K: Higher education associated with higher symptom recall (*x*^2^ [4] = 73.98, *p* < 0.001) B: Lower education associated with most negative beliefs (*x*^2^ [4] = 74.96, *p* < 0.001) SP: NS association with education and SP intentions (statistics NR)	Good
Meechan et al. (2003) [46]	Retrospective Mixed	85 women. Mean age: 38.9 years	New Zealand	Breast	Education	PB: Having a family member with cancer, low emotional response to symptom PF: High emotional response to symptom SP: Median time to symptom presentation (14 days)	PB: NR PF: NR SP: NS association between education and time to SP (t (83) = -1.26, *p* > 0.05)	Medium
Mor (1990) [74]	Retrospective Mixed	700 patients. Age range: 45 to 90 years	US	Lung, Breast and Colorectal	Education, housing, income, education	K: Symptom interpretation (best knowledge for breast cancer patients) B: Fear (16.8 % of delayers) PB: ‘’thought it would go away” (60.5 % of delayers), too busy (8.4 % of delayers) SP: Waited over 3 months to seek medical help: lung (54.9 %), breast (56.2 %), colorectal (87.6 %)	K: NR B: NR PB: NR SP: NS relationship between socioeconomic group and time to SP (statistics NR)	Medium
Oliveria et al. (1999) [37]	Retrospective Quantitative	255 men and women. Aged 18 years and over	US	Melanoma	Education, insurance	K: Recognition (poor) SP: Mean time to symptom presentation (2 months)	K: NR SP: Education not associated with time to SP (statistics NR)	Medium
O’Mahony and Hegarty (2009) [47]	Retrospective Quantitative	99 women. Mean age: 40 years	Ireland	Breast	Employment, education	K: Symptom interpretation PB: Competing life priorities, emotional reactions to symptom (afraid, scared, unsure) PF: Symptom disclosure, anxiety SP: Waited over 1 month to seek medical help (*n* = 26)	K: NR PB: NR PF: NR SP: Higher education associated with longer time to SP (statistics NR)	Medium
O’Mahony et al. (2011) [79]	Retrospective Qualitative	10 women. Mean age: 40 years	Ireland	Breast	Education, employment, insurance	K: Most aware that a lump was a symptomof cancer B: Fatalism, curability of cancer, fear PB: Denial, competing life priorities PF: Symptom disclosure, good perceived access to GP, good relationship with GP SP: Sought medical help within 1 month (*n* = 6)		Medium
Pedersen et al. (2011) [85]	Retrospective Quantitative	901 men (*n* = 423) and women (*n* = 487). Mean age: 61.8 years	Denmark	All	Education	PF: Symptom disclosure, good partner support SP: Median interval: 12 days	PF: NR SP: NS association between education and time to SP: Lower secondary education and long SP (>55 days) (RRR 0.79, 95 % CI: 0.36-1.74, *p* > 0.05); tertiary education and long SP (>55 days) (RRR 1.30, 95 % CI: 0.55-3.08, *p* > 0.05)	Medium
Quaife et al. (2014) [32]	Hypothetical Quantitative	6965 men (*n* = 4330) and women (*n* = 265). Aged 50 and over	UK	All	Education	K: Recognition (best for ‘lump’) PB: Poor access health services SP: Would wait 2+ weeks: (cough, *n* = 48.1 %; breast change, *n* = 8.2 %; rectal bleeding, *n* = 7.4 %)	K: Lower education associated with lower recognition for all 3 symptoms (*x*^2^, *p* < 0.05**) PB: NR SP: Lower education associated with shorter time to SP for cough (OR 0.61, 95 % CI: 0.54-0.68, *p* < 0.001) and breast changes (OR 0.68, 95 % CI: 0.52-0.89, *p* < 0.001). NS association with education and time to SP for rectal bleeding (OR 0.83, 95 % CI: 0.67-1.03, *p* > 0.05)	Good
Rauscher et al. (2010) [66]	Retrospective Quantitative	438 women. Age range: 30 to 79 years	US	Breast	Education, household income, health insurance status	K: Breast lump misconceptions (20 % reported one or more misconception) PB: Poor general health service utilisation SP: Waited over 3 months to seek medical help (16 %)	K: Lower income and education associated with more breast lump misconceptions (*x*^2^, *p* < 0.001**) PB: NR SP: Longer time to SP associated with lower education (*x*^2^, *p* < 0.05**) and lower income (*x*^2^, *p* < 0.05**)	Medium
Richard et al. (2000) [77]	Retrospective Quantitative	590 men (*n* = 250) and women (*n* = 340). Mean age: 51.2 years	France	Melanoma	Residence, social level, education	K: Symptom interpretation (‘not serious’, 34.8 %) B: Fear PB: No symptoms, competing life priorities (work and family commitments), melanoma not detected by participant PF: Active encouragement from family SP: Sought medical help within 2 months (51.9 %)	K: NS B: NS PB: Those with higher education more likely to self-detect melanoma (*x*^2^, *p* < 0.01**) PF: NR SP: NS association with and time to SP and socioeconomic group (statistics NR)	Medium
Rozniatowski et al. (2005) [73]	Retrospective Quantitative	100 men (*n* = 84) and women (*n* = 16). Mean age: 57 years	France	Head and Neck	Education, occupation	PB: Low anxiety, poor general health service utilisation PF: Symptom disclosure, active encouragement from partner to seek help SP: The majority of patients waited over 1 week to seek medical help	K: NR PB: NR SP: NS association between socioeconomic group and time to SP (statistics NR)	Medium
Ristvedt et al. (2014) [33]	Retrospective Quantitative	112 men (*n* = 55) and women (*n* = 57). Mean age: 59.3 years	US	Colorectal	Income, area of residence, education, health insurance	K: Symptom interpretation (70.5 % thought symptom serious within 13 weeks post onset) SP: Median time to symptom presentation (10 weeks)	K: NR SP: NS association between socioeconomic group (education and household income) and time to SP (statistics NR)	Medium
Ristvedt and Trinkhaus (2005) [9]	Retrospective Quantitative	69 men (*n* = 42) and women (*n* = 27). Mean age: 61.3 years	US	Colorectal	Education	K: Symptom interpretation (‘not cancer’, 71 %) PB: Personality (low trait anxiety), poor health service utilisation SP: Mean time to symptom presentation (25 weeks)	K: NR PB: NR SP: Lower education associated with longer time to SP (Kaplan-Meier: median 15 weeks, 95 % CI: 9.0-26.0*); higher education associated with shorter time to SP (Kaplan-Meier: median 8 weeks, 95 % CI: 4.0-15.0*)	Medium
Robb et al. (2009) [19]	Hypothetical Quantitative	2216 men (*n* = 968) and women (*n* = 1240)	UK	All	Education, occupation	K: Recall (poor, mean = 2.2) and recognition (good, mean = 7.2) PB: Emotional and service barriers most endorsed SP: Most would seek medical help within 2 weeks	K: Higher socioeconomic group (occupation) associated with highest knowledge (F (2,2015) = 20.31, *p* < 0.001) PB: Lower socioeconomic group (occupation) associated with more emotional barriers endorsed: ‘worry what the doctor might find’ (*x*^2^ (1,1989) = 17.08, *p* < 0.001), ‘too embarrassed’ (*x*^2^ (1,1993) = 20.74, *p* < 0.001), ‘not confident to talk about symptom’ (*x*^2^ (1,1992) = 4.77, *p* < 0.05), NS association with ‘too scared’ (*x*^2^ (1,1977) = 1.82, *p* > 0.05); Higher socioeconomic group (occupation) associated with more practical barriers endorsed: ‘too busy’ (*x*^2^ (1,2005) = 59.0, *p* < 0.001), ‘other things to worry about’ (*x*^2^(1,1996) = 15.34, *p* < 0.001), ‘difficult to arrange transport’ (*x*^2^(1,2010) = 11.13, *p* < 0.001); NS association between socioeconomic group (occupation) and service barriers: ‘difficult to make appointment’ (*x*^2^ (1,1983) = 0.41, *p* > 0.05), ‘worried about wasting the doctors time’ (*x*^2^ (1,1995) = 1.44, *p* > 0.05), ‘difficult to arrange transport’ (*x*^2^ (1,1938) = 1.15, *p* > 0.05) SP: Lower socioeconomic group (occupation) associated with shorter time to SP for unexplained bleeding (*x*^2^ (1,1991) = 5.82, *p* < 0.01), difficulty swallowing (*x*^2^ (1,1987) = 28.41, *p* < 0.001), lump (*x*^2^(1,1988) = 21.26, *p* < 0.001), change in mole (*x*^2^ (1,1967) = 24.24, *p* < 0.001), unexplained pain (*x*^2^(1,1965) = 20.24, *p* < 0.001), sore that does not heal (*x*^2^ (1,1977) = 35.84, *p* < 0.001), change in bowel/bladder habits (*x*^2^ (1,1982) = 56.87, *p* < 0.001), cough (*x*^2^ (1,1984) = 48.32, *p* < 0.001), unexplained weight loss (*x*^2^ (1,1963) = 77.73, *p* < 0.001)	Good
Samet et al. (1988) [62]	Retrospective Quantitative	800 men (*n* = 396) and women (*n* = 404). Mean age: 72.2 years	US	All	Education, income	PB: Poor general health service utilisation, poor access SP: Most sought medical help within 2 months	PB: NR SP: Longer time to SP associated with lower income for breast and colorectal cancer (*x*^2^, *p* < 0.05**) and lower education for all tumour sites (*x*^2^, *p* < 0.05**)	Medium
Schmid-Wendter (2002) [40]	Retrospective Quantitative	233 men (*n* = 109) and women (*n* = 109). Mean age: 54.5 years	Germany	Melanoma	Education	K: Previous knowledge of melanoma, symptom interpretation B: Fear PB: Lesion not visible, too busy SP: Sought medical help within 1 month (15.5 %)	K: Higher education more likely to have knowledge about melanoma (*x*^2^, *p* < 0.001**) B: NR PB: NR SP: NR	Medium
Siminoff et al. (2014) [35]	Retrospective Mixed methods	252 men (*n* = 132) and women (*n* = 120). Mean age: 58 years (range 25 to 94 years)	US	Colorectal	Education, Employment, Income	K: Symptom interpretation (39.7 % did not think symptom was serious) PB: Financial barriers (28.6 %), fear of diagnostic tests (24.3 %), embarrassment (11.9 %) SP: Mean appraisal delay (4.8 months)	K: NR PB: NR SP: NS association between time to SP and socioeconomic group (statistics NR)	Medium
Simon et al. (2010) [49]	Retrospective Quantitative	236 men (*n* = 968) and women (*n* = 1240). 11.4 % (*n* = 236) had experienced a symptom in the past 3 months	UK	All	Occupation	K: Recognition (better knowledge if experienced a symptom previously); symptom interpretation (worry symptom might be cancer) PB: Emotional and practical barriers SP: Symptom experience: 11.4 % experienced symptom in past 3 months (75 % consulted a GP about symptom)	K: NS association between symptom interpretation and socioeconomic group (statistics NR) PB: NR SP: NS association between SP and socioeconomic group (statistics NR)	Good
Smith and Anderson (1985) [51]	Retrospective Quantitative	82 women. Age range: 20 to 54 years	US	Ovarian	Income, education, occupation	K: Symptom interpretation (‘cancer’, 10 %) B: Fear PB: Previous benign diagnosis SP: Median time to symptom presentation (4 weeks)	K: NS association between symptom interpretation and socioeconomic group (statistics NR) B: NR PB: NR SP: NR	Medium
Temoshok et al. (1983) [75]	Retrospective Quantitative	106 men and women. Age range: 18 to 72 years.	US	Melanoma	Education, occupation	K: Previous knowledge of melanoma B: Melanoma not a serious disease PF: Lesion visible (face and neck) SP: Mean time to symptom presentation (4 months)	K: No association with knowledge and occupation (statistics NR) B: NR PF: NR SP: No association with time to SP and occupation (statistics NR)	Poor
Tod et al. (2008) [80]	Retrospective Qualitative	20 men (*n* = 12) and women (*n* = 8).	UK	Lung	Occupation	K: Symptom interpretation (poor, symptoms usually interpreted as acute conditions) B: Fear, fatalism PB: If previously given up smoking (thought risk of lung cancer was nil), worry about the wasting doctors time, previous bad experiences with health system, blame, stigma, stoicism, poor health service utilisation PF: Active encouragement from family member SP: Range in time to symptom presentation (0 to 24 months)		Good
Tomlinson et al. (2012) [60]	Retrospective Quantitative	87 men (*n* = 56) and women (*n* = 31). Mean age: 65 years.	Canada	Colorectal	Education	K: Symptom interpretation PB: Self medication SP: Waited over 1 month to seek medical help (51 %)	K: NR PB: NR SP: NS association between education and time to SP (*x*^2^, *p* > 0.05**)	Medium
Trivers et al. (2011) [52]	Hypothetical Quantitative	2991 women. 65 % were aged 45 years and over.	US	Gynaeco-logical	Education, Income	B: Concern about developing gynaecological cancer PB: Being premenopausal SP: 50 % of women would seek help for most symptoms	B: NR PB: NR SP: NS association between SP intentions and socioeconomic group (statistics NR)	Medium
Van Osch et al. (2007) [48]	Hypothetical Quantitative	459 men (49 %) and women (51 %) over the age of 55. Mean age: 68.6 years.	Netherlands	All	Education	K: Recognition (low to moderate, mean: 6.2) B: Benefits of early detection SP: Fair. Inconsistent for urgent symptoms, good for prolonged symptoms	K: NR B: NR SP: Lower education associated with shorter time to SP (F (2,436) =6.084, *p* < 0.01)	Good
Waller et al. (2009) [53]	Hypothetical Quantitative	1500 men and women from various ethnic minority groups.	England	All	Occupation	K: Recall (poor, mean: 1.2) and recognition (poor, mean: 4.7) PB: Worry what doctor might find (most endorsed) SP: African and Caribbean groups anticipated fastest time to symptom presentation	K: Higher socioeconomic group associated with higher recall (F(1,1487) = 6.12, *p* < 0.01) and higher recognition (F (1,1487) = 5.45, *p* < 0.05) PB: NR SP: NR	Good
Walter et al. (2014) [41]	Retrospective Qualitative	63 men (*n* = 31) and women (*n* = 32). Age range: 29-93 years.	UK	Melanoma	Education	K: Symptom attributions (initially attributed to benign skin conditions or normal life changes) PB: Worry about wasting the doctors time, service barriers, competing life priorities, reassurance following symptom disclosure PF: Family history of melanoma, perceptions of high risk, symptom disclosure, symptom noticed by another person SP: Range 1-303 weeks		Good
Whitaker et al. (2014) [55]	Retrospective Quantitative	1724 men (*n* = 789) and women (*n* = 921) over the age of 50. Mean age: 64.4 years.	England	All	Postcode, education, employment	K: Symptom interpretations (2 % thought symptom was cancer, highest interpretation for ‘unexplained lump’), perceived seriousness of symptoms SP: Symptom experience (53 % experienced at least 1 symptom in past 3 months). 59 % contacted GP about symptom	K: Unemployment associated with higher perceived seriousness of pain (OR 2.26, 95 % CI: 1.17-4.35, *p* < 0.05), tiredness (OR 2.11, 95 % CI:1.23-3.64, *p* < 0.05), sore throat (OR 3.56, 95 % CI: 1.10-11.45, *p* < 0.05) and chest pain (OR 3.56, 95 % CI: 1.10-11.45, *p* < 0.05). Lower education associated with higher perceived seriousness cough (OR 2.25, 95 % CI: 1.10-4.56, *p* < 0.05), tiredness (OR 2.46, 95 % CI:1.44-4.21, *p* < 0.05), headaches (OR 3.80, 95 % CI: 1.63-8.89, *p* < 0.05), shortness of breath (OR 2.34, 95 % CI: 1.11-4.97, *p* < 0.05), sore throat (OR 4.16, 95 % CI: 1.14-15.22, *p* < 0.05) and chest pain (OR 4.16, 95 % CI: 1.13-15.22, *p* < 0.05) SP: NR	Good
Whitaker et al. (2015) [81]	Retrospective Qualitative	48 men (*n* = 23) and women (*n* = 25) over the age of 50. Mean age: 64.4 years.	England	All	Education, employment	K: Symptom interpretations (symptoms normalised or associated with cancer) PB: Stoicism, fear of diagnostic tests, worry about wasting doctors time, service barriers, negative attitudes towards HCPs, medical mistrust PF: Development of new symptoms, persistence of symptoms, symptom disclosure, fear SP: Varied per symptom: 33.3 % contacted GP with ‘persistent cough’, 100 % contacted GP with ‘unexplained bleeding’		Good

*K* = cancer symptom knowledge; *B* = beliefs about cancer; *PB* = perceived barriers to symptom presentation; *PF* = perceived facilitators to symptom presentation; *SP* = time to symptom presentation; *NR* = not reported; *NS* = not significant; *p-value not reported; **other statistics not reported

### Symptom presentation

Studies involving anticipated symptom presentation reported shorter time to symptom presentation compared with studies that examined actual time to symptom presentation. In the former, most participants anticipated seeking medical help within one week [28–30] or within one month [19, 31, 32], in contrast to real-world studies where it was more common for patients to have waited over two months before seeking medical help [33–41]. The most prompt actual and anticipated symptom presentation was reported for lumps [32, 38, 42–47] or bleeding [19, 32, 48–53]. Studies examining participants who reported experiencing a potential symptom of cancer in the past three months found between 59 % and 75 % of participants had consulted a doctor about their symptom [49, 54, 55].

Disparity between actual and anticipated symptom presentation relating to socioeconomic group was observed. In five studies, shorter anticipated time to symptom presentation was observed in lower compared to higher socioeconomic groups [19, 28, 31, 32, 48]. Conversely, in two studies, longer anticipated time to symptom presentation was reported in those from lower socioeconomic groups compared with higher socioeconomic groups [56, 57].

Studies which measured actual time to symptom presentation reported the longest delays in symptom presentation among individuals with lower educational attainment [33, 34, 42, 54, 58–61], lower annual income [61, 62], lower occupation and employment [43, 61, 63] and those from deprived areas [64]. This effect was also observed in studies of actual symptom presentation where multiple socioeconomic indices were reported [34, 42, 44, 61, 65, 66]. Twenty-two studies found no group differences for socioeconomic group indicators and time to symptom presentation [30, 33, 35–37, 45, 46, 49, 50, 52, 60, 67–77].

### Knowledge

Knowledge of symptoms based on recall methods was generally lower than in studies that used recognition methods. Lump symptoms were the most recalled and well-recognised potential cancer symptom [19, 32, 48, 50, 53, 56, 64, 78]. This was supported by retrospective studies where patients presenting with a lump were most likely to have attributed their lump symptom to cancer [39, 43, 45, 74, 79]. Knowledge was generally poor for non-specific symptoms of cancer. Symptoms such as fatigue or unexplained weight loss were poorly recalled or recognised as potential symptoms of cancer [28, 29, 31, 53, 78]. Poorer cancer symptom knowledge was associated with lower socioeconomic group when measured by educational attainment [28, 32, 40, 50, 54, 56], occupation [53] and multiple indicators [19, 28, 66]. These findings were consistent across site-specific and non site-specific studies, suggesting poor general cancer symptom knowledge in lower socioeconomic groups regardless of cancer type.

In retrospective studies, patients experiencing non-specific symptoms recalled attributing them to other benign causes or life stresses [35, 51, 55, 65, 69, 76, 80, 81] or not recognising the seriousness of their symptoms [9, 33, 35, 37, 40, 42, 43, 45, 47, 51, 54, 55, 57, 60, 65, 68, 76, 77, 81, 82] resulting in patients delaying symptom presentation [35, 39, 51, 76] or later stage at diagnosis [69].

### Beliefs about cancer

In most studies, beliefs were formed from participants’ past experiences of cancer, usually witnessing friends or family with the disease [36, 43, 47, 59, 78, 79]. Positive beliefs were identified in nine studies [30, 36, 43, 48, 54, 58, 78, 79] and tended to focus on the effectiveness of modern cancer treatments, where participants expressed trust in doctors and the medical system and endorsed the benefits of early diagnosis [30, 58, 59, 78] or acknowledged that cancer can be cured [78]. Such beliefs tended to encourage timely symptom presentation to a primary care physician [30, 58, 59, 78, 79]. One study found that those with lower educational attainment were less likely to endorse positive beliefs about the benefits of early detection [54].

Negative beliefs tended to manifest in fear or fatalism regarding cancer. Fear was frequently reported across all studies examining beliefs. This included fear of diagnosis [34, 39, 58, 63, 74, 80, 81, 83], fear of treatment [30, 43, 57–59, 68, 78, 83] and fear of dying [59, 78, 83]. Fatalistic beliefs were a common theme throughout studies, but were expressed only by a minority of participants per study [34, 36, 42, 56, 59, 61, 78, 79, 84]. Fearful and fatalistic beliefs about cancer were more likely to be expressed by individuals from lower socioeconomic groups based on educational attainment [36, 50] or multiple indices [42, 71].

When considering time to symptom presentation, fearful beliefs about cancer appeared to operate at the two extremes of immediate or prolonged symptom presentation. For participants whose fearful beliefs encouraged immediate (actual or hypothetical) presentation to doctors [43, 45, 58, 59, 61, 74, 78, 79, 84], a visit to doctors was used to alleviate anxiety associated with the symptom [43, 47, 58, 59, 61, 77, 78]. This was usually coupled with the participant expressing trust in the medical profession and positive beliefs surrounding early diagnosis [43, 59].

For individuals whose fearful beliefs led to prolonged delays (sometimes years) [30, 34, 38, 39, 43, 47, 51, 61, 68, 74, 78, 79], denial of or ignoring symptoms initially alleviated anxiety associated with the symptom [38, 39, 47, 59, 68, 72, 76, 78, 79]. Such beliefs were usually combined with fatalistic beliefs such as ‘cancer cannot be cured’ [59, 61, 79], and were associated with the longest times to symptom presentation or were expressed by those with advanced stage disease [36, 56, 59, 84]. This is likely to reflect a lack of perceived benefit in presenting to doctors due to the belief that ‘nothing can be done’ [59, 78].

### Barriers to symptom presentation

Some participants reported service barriers relating to concerns about wasting doctors’ time [19, 29, 31, 34, 41, 43, 55, 80, 81], lack of continuity with primary care doctor [42, 81] or difficulties with accessing and making an appointment [29, 32, 34, 38, 53, 55, 56, 65, 78, 81]. For others, practical barriers such as being ‘too busy to make an appointment’ were reported and these delayed symptom presentation [31, 39, 40, 43, 49, 74, 77, 78]. Low general health service utilisation for acute or long term conditions lengthened time to cancer symptom presentation [9, 34, 42, 43, 57, 58, 61, 66, 68, 73, 77, 78, 80, 84]. Emotional barriers included embarrassment or fear associated with undergoing intimate diagnostic tests [19, 29, 31, 34, 35, 49, 57, 78, 81].

Practical barriers such as ‘being too busy’ were more frequently reported in high socioeconomic groups [19]. In countries where patients pay for their healthcare, those with lower annual income were more likely to report the cost of a consultation as a barrier to symptomatic presentation [38, 63].

### Facilitators to symptom presentation

The most common facilitator of symptom presentation was disclosure of symptoms to a family member or friend [34, 39, 41, 43, 45, 47, 55, 61, 65, 70, 73, 76–79, 81, 84, 85]. In some cases, this reduced time to symptom presentation by half [36] or by six times [45]. The appearance of a new symptom [43, 69, 76, 83] or persistence of the current symptom [45, 69, 76, 81, 84] facilitated decisions to seek medical help. One study found that individuals from a lower socioeconomic group who disclosed their symptom to a family member or friend took longer to seek medical help compared to those from a higher socioeconomic group [65]. In five studies, participants waited until they developed another health complaint or tagged their cancer symptom on to the end of a consultation which provided an opportunity to disclose the cancer symptom during the consultation [42, 45, 68, 81, 82].

## Discussion

This review is the first to systematically explore how knowledge, beliefs and barriers/facilitators to symptom presentation affect actual or anticipated cancer symptom presentation in relation to socioeconomic group, across all tumour sites. Poor knowledge of non-specific cancer symptoms such as fatigue and weight loss prolonged presentation due to misattribution of symptoms in lower socioeconomic groups. In contrast, lump and bleeding symptoms were most frequently recalled, recognised and prompted the fastest symptom presentation. A knowledge gradient was observed, where poorer cancer symptom knowledge was associated with lower socioeconomic group based on multiple indices. There was some evidence to suggest that those from a lower socioeconomic group were more likely to hold fearful and fatalistic beliefs about cancer and less likely to endorse positive beliefs about the benefits of early diagnosis. Such combinations of fearful and fatalistic beliefs were associated with prolonged symptom presentation. In addition, emotional barriers to symptom presentation such as worry what the doctor might find were more likely to be endorsed in lower socioeconomic groups. Such poor knowledge and prevalent beliefs might account for the long actual delays and later stage cancers diagnosed in lower socioeconomic groups. Disclosure of a symptom to a family member or friend was a key facilitator in the decision to seek medical help, although there was some evidence to suggest that symptom disclosure acted as a barrier in lower socioeconomic groups.

Most included studies were of medium quality. In many studies, socioeconomic group was measured but not reported for all outcome variables. Most studies only reported socioeconomic group differences for symptom presentation. Twenty-three studies reported socioeconomic group differences for the other outcome measures: knowledge, beliefs and barriers/facilitators to symptom presentation. A further eight studies could have met the inclusion criteria, but were excluded due to non-reporting of any outcomes associated with socioeconomic group [14, 86-92]. Methodological limitations included a long duration between cancer diagnosis and participation in retrospective studies, and samples biased towards higher socioeconomic groups. In some studies, socioeconomic variation was insufficient to perform statistical analysis on all outcomes.

There are methodological limitations associated with retrospective (actual symptom presentation) and hypothetical (anticipated symptom presentation) designs. Whilst retrospective studies are affected by recall bias, hypothetical studies rely on intentions which may not translate into actual presentation behaviour [93]. This was observed in the variation between actual and hypothetical time to symptom presentation, where participants anticipated prompt symptom presentation but in reality reported longer delays. Study designs exploring actual symptom presentation behaviour in a population sample are likely to reduce some of the limitations associated with retrospective and hypothetical symptom presentation study designs. In such study designs, participants disclose actual symptoms experienced in the past three months, usually prompted by a list (without any mention of cancer), and reasons for not consulting a doctor are explored [49, 54, 55, 81].

The limitations of this review include problems relating to retrieval of studies and analysis of the evidence. Due to poor indexing of studies in this topic area under the MeSH indexing in this topic area, a high proportion of studies (*n* = 11) was found through hand-searching. Additionally, meta-analysis was precluded by the wide range of qualitative and quantitative data collection methods of included studies. Finally, other factors such as age, gender and ethnicity can affect symptom presentation [6, 18]; however, interactions between these variables and socioeconomic group were not addressed in the current review.

The findings of the current review confirm that failure to appreciate the seriousness of symptoms [6, 16] and non-disclosure of symptoms [6, 15] lengthened time to symptom presentation. Our findings accord with previous studies in which negative beliefs [20], longer time to actual symptom presentation [6] and low suspicion for cancer symptoms [94] were associated with low socioeconomic group [6]. The current findings support Mitchell et al.*’*s (2008) [16] review of colorectal cancer patients, in which fear of cancer either lengthened or shortened time to symptom presentation. Such findings might be explained by Type I and Type II information processing systems. Type I processing is a fast and automatic system, which represents an individual’s ‘gut reaction’ to an event, whereas Type II is a slower, more thoughtful and deliberative system [95]. Whilst most people initially experience fear in reaction to a worrying symptom (Type I processing), cognitions during Type II processing may influence the decision to seek medical help since these are slower and may help someone to rationalise the situation [96]. If an individual has had time to consider the benefits of seeking medical help, and based upon their previous beliefs about early diagnosis, such beliefs may override the Type 1 fear response. We found evidence to suggest a higher prevalence of fearful and fatalistic beliefs in lower socioeconomic groups and some evidence for fewer positive beliefs surrounding the benefits of early diagnosis in lower socioeconomic groups. This suggests that Type I beliefs may not be overridden by Type 2 responses relating to the benefits of early diagnosis due to lower knowledge or higher emotive responses. As a consequence this may delay symptom presentation. Findings relating to symptom disclosure suggest that people use the ‘lay system’ of healthcare (consulting family and friends) before making the decision to access formal healthcare [13, 97, 98]. However, among individuals from low socioeconomic groups, disclosing symptoms to someone with equally poor knowledge and Type I negative automatic beliefs about cancer may encourage false reassurance in the benign nature of symptoms and consequently no urgency to seek medical help.

Cancer awareness interventions should be carefully developed to target those who are most likely to present with advanced stage disease: lower socioeconomic groups with low symptom knowledge and fearful and fatalistic beliefs about cancer. Such an intervention should utilise an individual’s social networks to facilitate distribution of information [97], highlighting the significance of non-lump symptoms as potentially indicative of cancer, along with advice on an appropriate time in which an individual should seek medical help and how to access such help [99]. This should be coupled with information outlining the benefits of early diagnosis and improved effectiveness of modern treatments for cancer, countering negative beliefs surrounding cancer. Future research should evaluate the effectiveness of such interventions in lower socioeconomic groups.

## Conclusion

Knowledge of potential cancer symptoms, beliefs about cancer and barriers to symptom presentation work in combination to influence symptom presentation: knowledge is necessary for accurate symptom appraisal, but beliefs about cancer and barriers to symptom presentation influence the decision to seek medical help or not. This is especially important in the context of socioeconomic deprivation, where lower knowledge, higher negative beliefs about cancer and perceived barriers may lead to avoidable delays, later stage of diagnosis and ultimately poorer survival outcomes. Targeted interventions should not only educate people about symptoms for cancer, but also work to break down unhelpful myths surrounding cancer survival and treatment options. They should address the barriers that people in lower socio-economic groups experience, and use social networks to raise awareness and support early symptom presentation.

## Additional file

Additional file 1: Appendix 1. Search terms. (DOCX 13 kb)

## Abbreviations

NAEDI: National awareness and early diagnosis initiative
SPIDER: Sample phenomenon of interest, design, evaluation, research type
CASP: Critical appraisal skills programme
OECD: Organisation for economic co-operation and development
