# Supplementary material for: Influences of cancer symptom knowledge, beliefs and barriers on cancer symptom presentation in relation to socioeconomic deprivation: a systematic review
Source: BMC Cancer. 2015 Dec 23;15:1000. doi: 10.1186/s12885-015-1972-8 (PMC4688960; doi:10.1186/s12885-015-1972-8)
Supplement: Additional file 1: Appendix 1. — Search terms. (DOCX 13 kb) [file 12885_2015_1972_MOESM1_ESM.docx]

Appendix 1: Search strategy

| SPIDER | Description | Equation used for search |
| --- | --- | --- |
| **Sample** | Adults (18+) male and female | **Sample: ‘**‘Adult’’ OR ‘’18 and over’’ OR ‘’over 18’’ OR ‘’male’’ OR ‘’female’’ OR ‘’deprived area(s)’’ OR ‘’socioeconomic status’’ OR ‘’deprived’’, OR ‘’NOT child’’ |
| **Phenomenon of Interest** | How cancer symptom knowledge, beliefs about cancer and barriers/facilitators to symptom presentation affect actual or anticipated cancer symptom presentation | **Cancer**: ‘’Cancer’’ OR ‘‘Malignancy’’ OR ‘’Tumour’’ OR ‘’Sarcoma’’ OR ‘’Melanoma’’ OR ‘’Disease’’  **Knowledge:** ‘’Awareness’’ OR ‘’Cancer awareness’’ OR ‘’knowledge’’ OR ‘’knowledge of cancer symptoms’’ OR ‘’passive detection’’ OR ‘’recognition’’ OR ‘’recall’’ OR ‘’appraisal’’ OR ‘’appraisal of symptom*’’  **Symptoms**: ‘’symptom’’ OR ‘’cancer symptom’’ OR ‘’cancer sign’’ OR ‘’sign’’ OR ‘’warning sign’’ OR ‘’early warning sign’’ OR ‘’suspected symptom*’’  **Beliefs:** ‘’beliefs’’ OR ‘’attitudes’’ OR ‘’thoughts’’ OR ‘’feelings’’ OR ‘’negative beliefs’’ ‘’negative attitudes’’ OR ‘’fear’’ OR ‘’anxiety’’ OR ‘’worry’’ OR ‘’concern’’ OR ‘’Embarrass*’’ OR ‘’fatalism’’ ‘’positive beliefs’’ OR ‘’positive attitudes’’ OR ‘’hope’’ OR ‘’optimism’’ OR ‘’benefit’’ OR ‘’spiritual’’  **Perceived barriers**: ‘’barriers to present*’’ OR ‘’service barrier*’’ OR ‘’emotional barrier*’’ OR ‘’practical barrier*’’  **Symptomatic presentation:** ‘’help seeking behaviour’’ OR ‘’help seeking’’ OR ‘’health seeking behaviour’’ OR ‘’health seeking’’ OR ‘’early presentation’’ OR ‘’late presentation’’ OR ‘’presentation’’ OR ‘’delay’’ OR ‘’patient delay’’ OR ‘’delay in help OR health seeking’’ OR ‘’behaviour’’ OR ‘’intentions’’ OR ‘’intentions to seek help’’ |
| **Design** | Both qualitative and quantitative methods | **Study design:** ‘’questionnaire’’ OR ‘’survey’’ OR ‘‘interview’’ OR ‘’focus group’’ OR ‘’case study’’ OR ‘’observ*’’ |
| **Evaluation** | Outcome measures. Cancer symptom knowledge, beliefs about cancer, barriers/facilitators to symptom presentation and symptom presentation. | **Outcome measures:** ‘’help seeking behaviour’’ OR ‘’experinec*’’ OR ‘’view’’ OR ‘’opinion’’ OR ‘’perce*’’ OR ‘’belie*’’ OR ‘’feel*’’ OR ‘’know*’’ OR ‘‘understand*’’ |
| **Research Type** | Qualitative, quantitative or mixed methods | **Methods:** ‘’qualitative’’ OR ‘’quantitative’’ OR ‘’mixed methods’’ |
